# Metabolic response by FDG-PET to imatinib correlates with exon 11 KIT mutation and predicts outcome in patients with mucosal melanoma

**DOI:** 10.1186/s40644-014-0030-0

**Published:** 2014-11-12

**Authors:** Katherine Zukotynski, Jeffrey T Yap, Anita Giobbie-Hurder, Jeffrey Weber, Rene Gonzalez, Thomas F Gajewski, Steven O’Day, Kevin Kim, F Stephen Hodi, Annick D Van den Abbeele

**Affiliations:** 1 Sunnybrook Health Sciences Centre, University of Toronto, 2075 Bayview Ave, Toronto M4N 3M5, ON, Canada; 2 Brigham and Women’s Hospital, 75 Francis Street, Boston 02115, MA, USA; 3 Department of Radiology, Huntsman Cancer Institute, University of Utah, 2000 Circle of Hope Dr, Salt Lake City 84112, UT, USA; 4 Dana-Farber Cancer Institute, 450 Brookline Ave, Boston 02215, MA, USA; 5 Moffitt Cancer Center, 12902 Magnolia Dr, Tampa 33612, FL, USA; 6 University of Colorado Cancer Center, 1665 Aurora Court, Aurora 80045, CO, USA; 7 University of Chicago, 5841 S. Maryland Ave, Chicago 60637, IL, USA; 8 Beverly Hills Cancer Center, 8900 Wilshire Blvd, Beverly Hills 90211, CA, USA; 9 California Pacific Medical Center, 45 Castro St, San Francisco 94114, CA, USA

**Keywords:** Melanoma, KIT mutation, 18F-FDG-PET/CT, Imatinib

## Abstract

**Background:**

In patients with metastatic melanoma and KIT amplifications and/or mutations, therapy with imatinib mesylate may prolong survival. ^18^F-labeled 2-fluoro-2-deoxy-D-glucose (^18^F-FDG) PET/CT may be used to assess metabolic response. We investigated associations of metabolic response, mutational status, progression-free survival and overall survival in this population.

**Methods:**

Baseline and 4-week follow-up ^18^F-FDG-PET/CT were evaluated in 17 patients with metastatic melanoma and KIT amplifications and/or mutations treated with imatinib in a multicenter phase II clinical trial. The maximum standardized uptake values (SUVmax) were measured in up to 10 lesions on each scan. Metabolic response was classified using modified EORTC criteria. Each patient had a diagnostic CT or MR at baseline, after 6 weeks of therapy and then at intervals of 2 months and anatomic response was classified using RECIST 1.0. Median follow-up was 9.8 months.

**Results:**

Partial metabolic response (PMR), stable metabolic disease (SMD) and progressive metabolic disease (PMD) was seen in 5 (29%), 5 (29%), and 7 (41%) patients respectively. Five patients (29%) had a KIT mutation in exon 11, four of whom (80%) had PMR while 1 (20%) had SMD. Twelve patients (71%) did not have a KIT mutation in exon 11, and only 1 (8%) had PMR, 4 (33%) had SMD and 7 (58%) had PMD. There was agreement of metabolic and anatomic classification in 12 of 17 patients (71%). Four of 17 patients (24%) had PR on both metabolic and anatomic imaging and all had a KIT mutation in exon 11. Survival of patients with PMD was lower than with SMD or PMR.

**Conclusions:**

Metabolic response by ^18^F-FDG-PET/CT is associated with mutational status in metastatic melanoma patients treated with imatinib. ^18^F-FDG-PET/CT may be a predictor of outcome, although a larger study is needed to verify this.

**Clinical trial registration:**

NCT00424515

## Background

Malignant melanoma is a spectrum of diseases associated with several genetic aberrations. Mucosal and acral melanoma arising from sites such as the palms, soles and nail beds as well as cutaneous melanoma arising from chronically sun-damaged skin are forms of the disease with different but overlapping oncogenes and biologic behavior [1]-[5] that have recently been found to harbor KIT mutations [6]-[10]. The majority of these mutations occur in exon 11 at the juxtamembrane portion of the protein, which predicts responsiveness to imatinib mesylate [11].

Imatinib mesylate (Gleevec) is a protein-tyrosine kinase inhibitor that inhibits platelet-derived growth factor and stem cell factor mediated cellular processes. It can also inhibit proliferation and induce apoptosis in Bcr-Abl positive cells or cells with a KIT mutation including melanoma [12]. In the past, imatinib mesylate has been successfully used to treat patients with gastrointestinal stromal tumor (GIST), where malignancy is associated with a KIT mutation [13]-[15]. Imaging in patients with GIST often includes ^18^F-labeled 2-fluoro-2-deoxy-D-glucose (^18^F-FDG) positron emission tomography/computed tomography (PET/CT), which can show metabolically active disease and may be more effective for evaluating therapeutic response to tyrosine kinase inhibition than anatomic imaging [16]-[20]. Further, changes in tumor metabolism may predict outcome.

In GIST, the majority of KIT mutations occur in exon 11 near the N-terminus. In contrast, melanoma patients have a higher prevalence of KIT mutations in exon 11 near the C-terminus as well as KIT mutations in exon 13 and exon 17, which may confer resistance to imatinib therapy [7]. Although preliminary results in the literature suggest imatinib therapy may be helpful for the treatment of melanoma [21], this may not be the case for all patients with melanoma despite the presence of a KIT mutation. In particular, recent studies by Woodman et al. and Antonescu et al. suggest that the L576P mutation in exon 11, the most common KIT mutation in melanoma, may induce structural changes resulting in resistance to imatinib therapy compared with other tyrosine kinase inhibitors such as dasatinib [22]-[24].

We previously suggested that imatinib can be effective when melanoma tumors harbor KIT mutations [25]. The aim of this study was to provide a detailed sub-analysis of the ^18^F-FDG-PET/CT data acquired during the course of the previously reported clinical trial [25]. Specifically, our aim was to assess the metabolic response of patients with metastatic melanoma and a KIT mutation and/or amplification treated with imatinib using ^18^F-FDG-PET/CT and to investigate the association of ^18^F-FDG uptake with mutational status, time-to-progression (TTP) and overall survival (OS).

## Methods

### Study description

The institutional review boards of the sites participating in a phase II multicenter clinical trial of imatinib in metastatic melanoma with KIT amplifications and/or mutations approved the study before patient enrollment and continuing approval was maintained throughout the study (This clinical trial was approved by the Dana-Farber/Harvard Cancer Center IRB).

Prior to enrollment in the trial, KIT mutational status was determined by tumor biopsy, followed by polymerase chain reaction, high performance liquid chromatography and DNA sequencing, with amplicons arising from exons 9, 11, 13 and 17. KIT gene amplification was assessed by quantitative polymerase chain reaction, where the threshold for increased KIT copy number relative to normal tissue was established using the 95% confidence level according to Chebychev’s inequality and the threshold for positive was 5.29 copies of KIT relative to GAPDH. KIT gene copy number was measured in relationship to a chromosome 2 peri-centromeric probe, corresponding to a region rarely subject to gain or loss in melanoma. A sample (or histologically distinctive region) was scored as amplified if the ratio of probe/centromere was >1.5 (highly-amplified >5.0). Oncogene mutation screened was also done on pre-treatment tissue in all patients using a mass spectroscopy system (Sequenom, San Diego, CA) and a panel of 643 hotspot mutations across 53 cancer genes.

Seventeen patients had baseline and follow-up ^18^F-FDG-PET/CT after one month of therapy. Metabolic tumor response was classified using modified European Organisation for Research and Treatment of Cancer (EORTC) criteria [26]. Anatomic tumor response was classified according to the best response achieved using Response Evaluation Criteria in Solid Tumors (RECIST) 1.0 applied to diagnostic CT or MR images obtained after 6 weeks of therapy and then at intervals of 2 months. The clinical trial methods have been previously described [25]. Choi criteria was not performed as the CT scans were acquired according to each institution’s standard of care in this multicenter trial with no standardization of the dosage and timing of contrast media [17].

### PET/CT acquisition and analysis

Patients fasted for 6 hours prior to the ^18^F-FDG-PET/CT. Each ^18^F-FDG-PET/CT was acquired approximately 60 minutes (mean: 62 min, range: 56 min – 70 min) after the intravenous administration of approximately 20 mCi ^18^F-FDG (mean: 20.1 mCi, range 17.6 mCi – 22.1 mCi). Whole-body imaging was performed from the skull vertex through the toes using a combined PET/CT scanner with images corrected for detector efficiency, attenuation, scatter, decay and random coincidences. Consistency was maintained across participating institutions by adherence to a standard quality assurance program at each site in accordance with NCI consensus guidelines [27]. All ^18^F-FDG-PET/CT images acquired were transferred to the Dana-Farber Cancer Institute after de-identification for central quality control and analysis. Two nuclear medicine physicians evaluated each study for extent and change of ^18^F-FDG-avid disease without knowledge of the mutational analysis, RECIST classification, or outcome. Final results were based on consensus. For each subject, up to 10 target lesions with the greatest FDG-avidity were identified on the baseline scans and analyzed on both the baseline and follow-up studies. Tumor FDG uptake was quantified using the maximum standardized uptake value (SUVmax). For each subject, the SUVmax of all target lesions was summed at each time point and the relative change in the summed SUVmax was used to assess metabolic response where: partial metabolic response (PMR) ≤ −25% < stable metabolic disease (SMD) < +25% ≤ progressive metabolic disease (PMD). The appearance of a new FDG-avid lesion in the post-treatment scans supersedes the relative change and results in PMD. Conversely, the complete resolution of all FDG-avid lesions in the post-treatment scans results in complete metabolic response (CMR).

### Statistical methods

The best overall response rate was defined as the proportion of patients with at least partial response as best response to therapy. The disease-control rate was defined as the proportion of patients who achieved complete response, partial response, or stable disease lasting at least 12 weeks as their best response to therapy. The agreement of response- and disease-control rate classifications based on metabolic response and RECIST 1.0 best overall response was conducted using McNemar’s test. The relationship between overall metabolic response and mutational status was explored using Fisher’s exact test. As there was not sufficient documentation of the location of biopsied lesions in this multi-center study, the overall metabolic response of the patient based on all lesions was compared to mutational status rather than the individual response of the biopsied lesion.

Time-to-progression (TTP) was defined as the time interval from study enrollment to the date of first documented disease progression. The follow-up of patients who died without disease progression was censored at the date of death; follow-up of patients who had not progressed at the time of the analysis was censored at the date of the last study assessment. Overall survival (OS) was defined as the time from study enrollment to death from any cause. The follow-up of patients who were alive at the time of data analysis was censored at the last date of vital status.

The distributions of TTP and OS are presented using the method of Kaplan-Meier, with point-wise, 95% confidence intervals estimated using log(−log(survival)) methodology. Comparisons of TTP and OS according to type of melanoma or mutational status were made using the log-rank test. To avoid bias, comparisons of TTP and OS according to metabolic response classification were conducted using one-month conditional landmark analyses [28]. Patients who were alive and progression-free at the time of the one-month metabolic tumor assessment were followed forward in time; patients with rapid anatomic progression of disease within one month were removed. Thus, 3 patients with rapid, anatomic progression prior to the one-month ^18^F-FDG-PET/CT were removed from this portion of the analysis. TTP and OS were then compared according to metabolic response categories using the log-rank test. Statistical significance was defined as p < 0.05; there were no corrections for multiple comparisons.

## Results

Nine medical centers participated in the phase II clinical trial between July 6, 2006 and March 1, 2011. Seventeen patients had baseline and follow-up ^18^F-FDG-PET/CT. Of the 17 patients with ^18^F-FDG-PET/CT included in the study, 7/17 (41%) had a KIT mutation, 5 of which were in exon 11 and 2 of which were in exon 13 or 17, while 10/17 (59%) had KIT amplification (Table 1). When there was both a KIT mutation and amplification, patients were classified as having a mutation for the purposes of analysis. At the time of enrollment, all patients had metastatic disease and a history of cancer-directed surgery. The median TTP was 4.7 months (95% CI: 1.3 to 7.4 months) and was independent of melanoma type (log-rank p = 0.98). TTP was similar for patients with a KIT mutation, or amplification (log-rank p = 0.33). Median OS was 12.9 months for patients with a KIT mutation in exon 11 (95% CI: 5.5 to ∞), 11.7 months for patients with a KIT gene amplification (95% CI: 2.8 to 16.2) and 10.4 months for patients with another type of mutation (95% CI: 1.5 to 19.3). Interestingly, the one patient with a KIT mutation in exon 17 did comparatively well with OS of 19.3 months (Table 1) while the 3 patients with the most rapid progression had amplification (TTP 1.0 and 0.9 months) and a KIT mutation in exon 13 (TTP 0.9 months).

**Table 1:** Patient characteristics and response assessment

**Sex**	**Age (yrs)**	**Genetic status**	**TTP (mo)**	**Survival (mo)**	**Metabolic response (modified EORTC)**	**Anatomic response (RECIST 1.0)**
Female	79	Exon 11 insertion PYD577-582	4.7	12.9	PMR	PR
Female	61	Exon 17 D820Y	10.2	19. 3	SMD	PR
Female	69	Amplified	7.4	8.8	SMD	PD
Male	57	Amplified	5.0	11.5	PMD	PD
Female	62	Amplified	2.6	11.9	SMD	PD
Male	69	Amplified	5.8	16.2	PMR	PD
Female	81	Amplified	1.0	3.8	PMD	PD
Female	72	Amplified	1.3	9.8	PMD	PD
Female	55	Amplified	10.6	18.3	SMD	SD
Female	59	Amplified	0.9	2.8	PMD	PD
Female	59	Exon 11 L576P	27.1	27.1	PMR	PR
Female	75	Amplified	5.6	12.5	PMD	SD
Female	47	Exon 11 L576P	2.6	7.3	PMR	PR
Male	84	Amplified	1.6	21.8	PMD	PD
Female	53	Exon 11 deletion WKVVE557-560	10.6	24.3	SMD	SD
Male	66	Exon 13 K642E	0.9	1.5	PMD	PD
Female	66	Exon 11 L576P	3.4	5.5	PMR	PR

PMR = Partial Metabolic Response, SMD = Stable Metabolic Disease, PMD = Progressive Metabolic Disease, PR = Partial Response, SD = Stable Disease, PD = Progressive Disease, TTP = Time to Progression.

**Figure 1:**
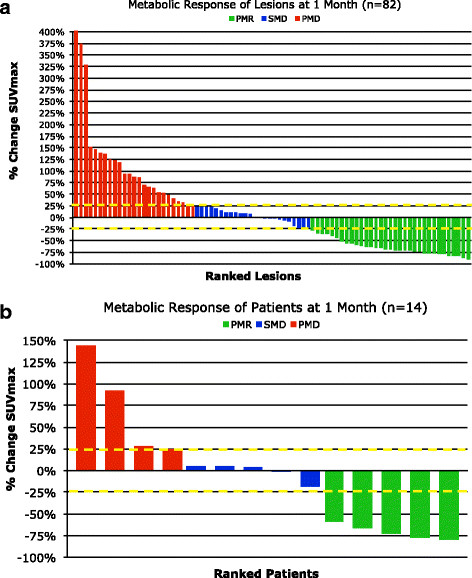
**Waterfall plots of metabolic response by lesion (a) and patient (b).** Three subjects were classified as PMD based on the presence of new lesions rather than % change SUVmax & have not been included in the waterfall plot above.

**Figure 2:**
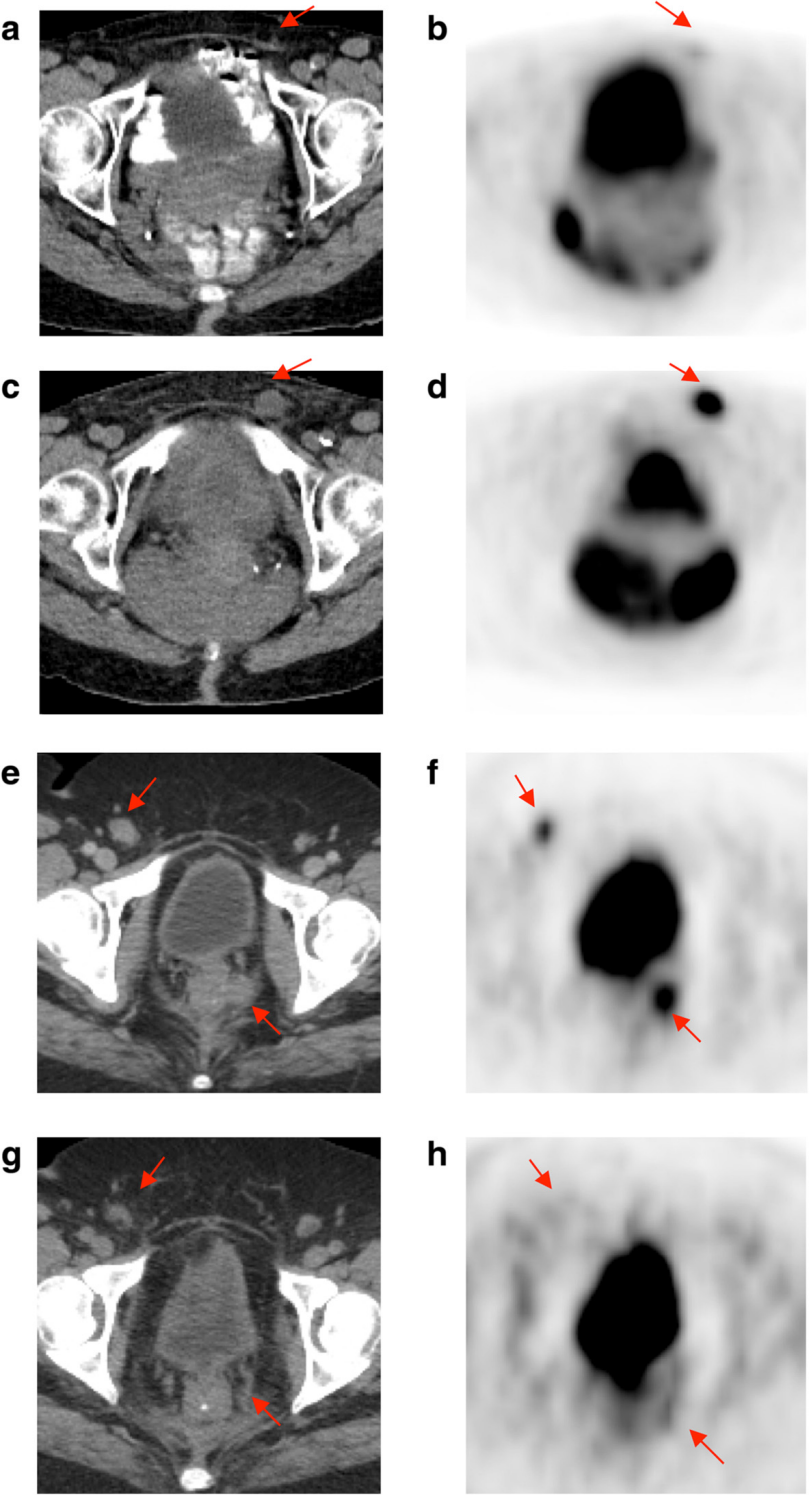
**Representative**^**18**^**F-FDG-PET/CT images showing progression in a patient without an exon 11 KIT mutation (a-d) and response in a patient with an exon 11 KIT mutation (e-h) at baseline (a-b, e-f) and after 1 month of therapy (c-d, g-h).** Red arrows point to sites of disease. Reprinted with permission. © (2013) American Society of Clinical Oncology. All rights reserved. From: Hodi S, et al. J Clin Oncol. 31(26), 2013:3182-3190.

Figure 1 shows waterfall plots of metabolic response by lesion and patient using ^18^F-FDG-PET/CT. PMR was seen in 5 of 17 patients (29%), SMD was seen in 5 of 17 patients (29%), and PMD was seen in 7 of 17 patients (41%). Figure 2 shows illustrative images of a patient with both PMR and anatomic PR compared with a patient with both PMD and anatomic PD after 1 month of therapy. There was agreement of metabolic and anatomic response classification in 12 of 17 patients (71%, 95% CI: 44% to 90%). For the 5 patients where metabolic and anatomic classification did not agree, the response to therapy using RECIST 1.0 and anatomic imaging after 6 weeks of therapy was slightly more likely to show PD than metabolic imaging obtained after 4 weeks of therapy.

**Figure 3:**
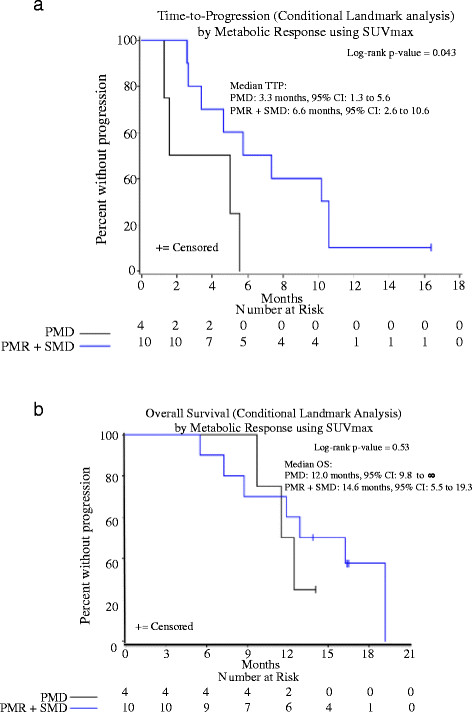
Time-to-progression (a) and overall survival (b) by metabolic response (Met).

Figure 3 shows the conditional landmark analyses of TTP and OS by metabolic response. Patients classified with PMR or SMD had significantly longer TTP than those with PMD (6.6 months vs. 3.3 months; log-rank p = 0.04). However, there was no evidence of a difference in overall survival (log-rank p = 0.53).

There was an association between metabolic response and KIT mutational status (Fisher’s exact p-value = 0.03). Five of 17 patients (29%, 95% CI: 10% to 56%) had a KIT mutation in exon 11. Of these 5 patients, 4 (80%) had PMR and 1 (20%) had SMD. Twelve of 17 patients (71%) did not have a KIT mutation in exon 11 (2 with other mutation and 10 KIT amplified), and only 1 (8%) had PMR, 4 (33%) had SMD and 7 (58%) had PMD. Four of 17 patients (24%) had PMR and PR with anatomic imaging and all of these patients had a KIT mutation in exon 11. At the time of the data analysis, 4 of 17 patients remained alive and 2 of them (50%) had a KIT mutation in exon 11.

## Discussion

In 2013, it is estimated that 76,690 new cases of malignant melanoma were diagnosed in the United States and there were 9,480 deaths from the disease [29]. Since prognosis depends on disease histology and the extent of metastases, among other factors, anatomic and metabolic imaging play a key role in patient evaluation. Specifically, ultrasound and lymphoscintigraphy are helpful for the evaluation of lymph node involvement [30],[31]. Diagnostic CT is used for the detection of metastases particularly to the lungs, lymph nodes and liver while MRI is used to evaluate the brain, meninges and spinal cord as well as to clarify indeterminate CT lesions [32]. The role of ^18^F-FDG-PET/CT has evolved in recent years. ^18^F-FDG-PET/CT is more accurate than anatomic imaging alone for the detection of metastases [32]-[35], and can lead to change in patient management [36],[37]. Surgical excision may be curative for disease confined to a thin lesion (<1 mm); however the prognosis for patients with metastases remains dismal [38].

There are few treatment options available for patients with metastatic melanoma [39]-[42]. Recently, immunotherapy has shown promising results. In particular, ipilimumab, a human monoclonal antibody that blocks cytotoxic T lymphocyte-associated antigen 4 (CTLA-4), has been associated with improved survival in patients with metastatic melanoma [43]. Targeted therapy for patients with specific oncogenic mutations including BRAF and NRAS has also been used. For example, vemurafenib, a selective inhibitor of the BRAF serine-threonine kinase enzyme has been linked to improved rates of overall survival in patients with the BRAF V600E mutation [44]. KIT mutations have been found in the rare subgroup of patients with mucosal melanoma, acral melanoma and melanoma arising on chronically sun damaged skin, which has raised the possibility of utilizing imatinib mesylate for targeted therapy in these types of melanoma.

Imatinib mesylate is a tyrosine kinase inhibitor that has previously been used to treat patients with GIST [14],[15]. These patients have a spectrum of neoplastic disease ranging from benign to malignant, depending on the tumor size, mitotic activity, anatomic site of origin and the presence of a KIT mutation [45]. It is thought that the KIT mutation promotes activation of tyrosine kinase and results in tumorigenesis [13]. Unfortunately, patients often develop secondary resistance to imatinib mesylate within 2 years of treatment, likely due to the presence of new KIT mutations in existing tumor cells. Although ^18^F-FDG-PET/CT is not used to make the initial diagnosis, it is helpful for monitoring therapy response and can detect therapy resistant tumors by demonstrating re-emergence of glycolytic activity and resulting increased FDG uptake. ^18^F-FDG-PET/CT has been used to evaluate response to molecular targeted therapy in several cancers including melanoma [46].

In our study, the median TTP for patients with melanoma and a KIT genetic aberration (mutation or amplification) was 4.7 months (95% CI: 1.3 to 7.4 months) and was independent of melanoma type (log-rank p = 0.98) or KIT aberration sub-type (log-rank p = 0.20). On ^18^F-FDG-PET/CT there was a spectrum of disease response to therapy at 1 month, as shown in Figure 1. Five of 17 patients (29%) responded to therapy, 5 of 17 patients (29%) had stable disease and 7 of 17 patients (41%) showed disease progression. As shown in Figure 3, the metabolic response to therapy on ^18^F-FDG-PET/CT could be used to detect therapy-resistant tumors. Specifically, patients with progressive metabolic disease on ^18^F-FDG-PET/CT had a median TTP on anatomic imaging that was approximately 3.3 months shorter than patients with SMD or PMR (log-rank p-value = 0.04). Further, the overall survival of patients with progressive disease on ^18^F-FDG-PET/CT was approximately 3 months shorter than patients with SMD or PMR. This modest improvement in overall survival following metabolic response to therapy may be due, in part, to secondary drug resistance.

Tumor metabolic change on ^18^F-FDG-PET/CT was also associated with KIT mutational status. The majority of patients with a KIT mutation in exon 11 had PMR while the majority of patients without a KIT mutation in exon 11 had PMD. All of the patients with both PMR and anatomic PR had a KIT mutation in exon 11.

The principal limitation of this study is the sample size. Only 17 patients had evaluable ^18^F-FDG-PET/CT at baseline and after 1 month of therapy and only 5 patients had a KIT mutation in exon 11. Three of 5 patients with a KIT mutation (60%) had the L576P mutation, which the literature suggests may have only limited response to imatinib therapy. Although 12 patients did not have a KIT mutation in exon 11, several of these patients had KIT amplification. The overall survival was only slightly lower for patients with KIT amplification compared with patients who had a KIT mutation. Nevertheless, our results suggest a significant correlation between metabolic response and exon 11 KIT mutation and suggest that metabolic change on ^18^F-FDG-PET/CT may detect therapy-resistant disease. Evaluation of a larger patient population will be needed to confirm the statistical significance of the parameters we have studied.

## Conclusions

Metabolic response by ^18^F-FDG-PET/CT is associated with exon 11 KIT mutational status in patients with mucosal melanoma, acral melanoma or melanoma arising on chronically sun damaged skin treated with imatinib. ^18^F-FDG-PET/CT may be a predictor of clinical outcome, although a larger study is needed to verify this.

## Competing interests

*The following authors have held consultant or advisory roles with the companies listed:* F. Stephen Hodi, Novartis; Jeffrey S. Weber, Novartis; Thomas F. Gajewski, Bristol-Myers Squibb, Roche/Genentech, GlaxoSmithKline, Abbvie, Jounce Therapeutics.

*The following authors have received research funding from the companies listed:* F. Stephen Hodi, Novartis, Pfizer; Rene Gonzalez, Novartis; Thomas F. Gajewski, Bristol-Myers Squibb, Roche/Genentech, Eisai, Merck, Incyte; Steven J. O’Day, Novartis; Kevin B. Kim, Novartis; Jeffrey T. Yap, Novartis; Annick D. Van den Abbeele, Novartis.

## Authors’ contributions

KZ contributed to the concept and design of the study, analysis and interpretation of data, and drafted and revised the manuscript, has given final approval of the version to be published, and agrees to be accountable for all aspects of the work. JY contributed to the concept and design of the study, analysis and interpretation of data, and revised the manuscript, has given final approval of the version to be published, and agrees to be accountable for all aspects of the work. AGH contributed to the concept and design of the study, analysis and interpretation of data, and revised the manuscript, has given final approval of the version to be published, and agrees to be accountable for all aspects of the work. JW contributed to the concept and design of the study, acquisition and interpretation of data, and revised the manuscript, has given final approval of the version to be published, and agrees to be accountable for all aspects of the work. RG contributed to the concept and design of the study, acquisition and interpretation of data, and revised the manuscript, has given final approval of the version to be published, and agrees to be accountable for all aspects of the work. TG contributed to the concept and design of the study, acquisition and interpretation of data, and revised the manuscript, has given final approval of the version to be published, and agrees to be accountable for all aspects of the work. SO contributed to the concept and design of the study, acquisition and interpretation of data, and revised the manuscript, has given final approval of the version to be published, and agrees to be accountable for all aspects of the work. KK contributed to the concept and design of the study, acquisition and interpretation of data, and revised the manuscript, has given final approval of the version to be published, and agrees to be accountable for all aspects of the work. SH contributed to the concept and design of the study, acquisition and interpretation of data, and revised the manuscript, has given final approval of the version to be published, and agrees to be accountable for all aspects of the work. AVDA contributed to the concept and design of the study, analysis and interpretation of data, and revised the manuscript, has given final approval of the version to be published, and agrees to be accountable for all aspects of the work. All authors read and approved the final manuscript.

## Acknowledgements

We are extremely grateful to patients who participated in the trial and their families for contributing their time and effort. We would like to thank Jill Berkowitz, Leonid Syrkin, Tricia Locascio, Amanda Abbott and all those in the research teams who worked to make this paper possible.
